# Manifold hepatoprotective actions of α-lipoic acid on metabolic function through redox regulation, inflammatory modulation, and anti-apoptosis after chronic sleep-deprived injury

**DOI:** 10.3389/fnut.2025.1679494

**Published:** 2026-01-22

**Authors:** Hung-Ming Chang, Hsing-Chun Lin, Ting-Yi Renn, Yu-Cheng Liu, Kai-Jung Yen, Chih-Kai Liao, Maria A. Tikhonova, Tamara G. Amstislavskaya, Sandeep Kumar Singh, Li-You Chen

**Affiliations:** 1 Department of Anatomy and Cell Biology, School of Medicine, College of Medicine, Taipei Medical University, Taipei, Taiwan; 2 Cell Physiology and Molecular Image Research Center, Wan Fang Hospital, Taipei Medical University, Taipei, Taiwan; 3 Department of Nutrition, Chung Shan Medical University, Taichung, Taiwan; 4 Department of Nutrition, Chung Shan Medical University Hospital, Taichung, Taiwan; 5 Master’s Program of Electro-Acoustics, Feng Chia University, Taichung, Taiwan; 6 Department of Anatomy, School of Medicine, College of Medicine, Chung Shan Medical University, Taichung, Taiwan; 7 Scientific Research Institute of Neurosciences and Medicine (SRINM), Novosibirsk, Russia; 8 Department of Medical Biotechnology, All India Institute of Medical Sciences, Nagpur, India; 9 Department of Medical Education, Chung Shan Medical University Hospital, Taichung, Taiwan

**Keywords:** alpha lipoic acid, chronic sleep deprivation, metabolic function, antioxidant, anti-inflammatory activity

## Abstract

**Objective:**

Chronic sleep deprivation (CSD) is a major public health issue that causes metabolic dysfunction by disrupting redox homeostasis and triggering hepatic inflammation. Given the strong anti-oxidative and anti-inflammatory properties of alpha lipoic acid (ALA), this study aims to determine whether exogenous ALA supplementation would provide hepatoprotection and therefore, maintain the metabolic activity following CSD.

**Methods:**

Adult male Wistar rats were randomly assigned to three groups (*n* = 6/group). The first two groups underwent three cycles of 5-day total sleep deprivation followed by 2-day rest, receiving daily 2 mL olive oil (CSD group) or ALA (100 mg/kg in 2 mL olive oil) (CSD + ALA group), respectively. The third group received no treatment serving as yoked control (CSC group). Spectrometric, biochemical, immunoblot, and immunohistochemical analyses were performed to assess the hepatic redox-active Fe^2+^ intensity, metabolic function, anti-oxidative enzyme expression, and the pro-inflammatory cytokine levels.

**Results:**

ALA significantly improves metabolic function as effectively attenuates serum concentrations of AST, ALT, and ALP, and stabilize hepatic Fe^2+^ intensity. ALA also remarkably decreases the protein level of inflammatory factors including NF-κB, TNF-α, IL-6, IL-1β, and cyclooxygenase-2 (COX2), as well as increases the expression of Nrf2-induced antioxidative enzymes such as SOD1 and glutathione peroxidase (GPx). Moreover, ALA further suppresses apoptosis and ferroptosis by modulating related markers including BAX, Bcl-2, GPx4, and FACL4 activities.

**Conclusion:**

These findings clearly demonstrate that ALA confers manifold hepatoprotective effects against CSD-induced dysfunction by significantly restoring redox balance, decreasing hepatic inflammation, suppressing apoptotic and ferroptotic signaling, thereby successfully preserving metabolic activity.

## Introduction

With the coming of the modern society, chronic sleep loss or sleep deprivation (CSD) has gradually become a prevalent and severe public health problem that affected millions of people worldwide (1, 2). According to the survey by the World Health Organization, there are nearly one-fifth populations suffered from extreme sleep problem that are strongly linked to poorer general wellbeing, and numerous psychiatric or physiological comorbidities (3). Previous studies have indicated that CSD would lead to increased incidences of cardiovascular morbidity, increased chances of metabolic dysfunction, development of neurodegenerative disorders, and promote the pathogenesis of several infectious or immuno-related deficiencies (4–7). Biochemical reports also demonstrated that enhanced oxidative stress, dysregulated apoptosis, and extensive inflammatory reactions mediated by overexpressed inflammatory factors [such as tumor necrosis factor α (TNF-α), interleukin 1β (IL-1β), and interleukin 6 (IL-6)] were served as the underlying mechanisms for the occurrence of various CSD-relevant diseases (8–10). Through DNA damage, lipid peroxidation, protein oxidation, and disturbed regulation of pro-apoptotic and anti-apoptotic factors [i.e. Bcl-2–associated X protein (BAX) and B-cell lymphoma 2 (Bcl-2), respectively], CSD would disrupt cellular membrane integrity and alter the structural proteins of ion channels, leading to tissue breakdown and further exaggerate the inflammatory response (8–10). Chronic and persistent inflammation would not only impair the cellular bioenergetics, but also contributes to the progression of systemic metabolic disorders (11). Given that enhanced oxidative stress, excessive apoptosis, and extensive inflammatory reaction play an essential role in the development of CSD-induced metabolic dysfunction, exogenous supplementation with anti-oxidative or anti-inflammatory agents may be worthy of trial for dietary use as an effective strategy to depress the metabolic dysfunction and improved the quality of life for those suffering from CSD.

Alpha-lipoic acid (ALA) is a naturally occurring organosulfur compound that functions as an effective anti-oxidative and anti-inflammatory agent (12). Due to its amphipathic nature, ALA could exert the protective effects in both aqueous and lipid environments, enabling it to scavenge a broad spectrum of reactive oxygen species (ROS) (13). Through directly neutralizing the free radicals, regenerating other endogenous antioxidants (such as glutathione, vitamins C and E, and coenzyme Q10), and chelating the redox-active metal ions (e.g., Fe^2+^), ALA could mitigate the Fenton reaction-mediated oxidative damage, thereby amplifying its powerful anti-oxidative activity (13–15). In addition to its potent anti-oxidative activity, ALA has also been demonstrated to possess significant anti-inflammatory action through inhibiting the nuclear factor kappa B (NF-κB) signaling pathway, resulting in the decreased production of pro-inflammatory cytokines (i.e., TNF-α, IL-1β, and IL-6) (16, 17). Moreover, ALA has further been reported to be capable of supporting the mitochondrial function by acting as a cofactor for key metabolic enzymes, leading to reduced mitochondrial ROS production and maintaining cellular bioenergetics (18, 19). These multifaceted bioactivities significantly highlight ALA’s therapeutic potential, particularly in counteracting oxidative stress and inflammation-related metabolic deficiency (20).

However, although the beneficial effects of ALA on protecting the metabolic function have been well documented, its protective role in the liver—the central organ in metabolic regulation and particularly susceptible to oxidative damage—following CSD has never been reported. Moreover, the detailed mechanism(s) participated in the hepatoprotective action of ALA on depressing the CSD-induced injury still remains to be further explored. As attempts to answer these questions, the present study is firstly aimed to determine whether the key hepatic enzymes and the redox-active metal ion involved in metabolic regulation [such as aspartate aminotransferase (AST), alanine aminotransferase (ALT), alkaline phosphatase (ALP), and the ferrous ion (Fe^2+^)] would significantly be improved in animals suffered from CSD and received ALA treatment. Secondly, this study further designed to examine whether ALA treatment would successfully preserve the liver and metabolic function after CSD through its manifold hepatoprotective actions, namely, depressing the oxidative stress, reducing the extensive inflammatory reaction, and inhibiting the apoptotic signaling activity.

## Materials and methods

### Experimental animals

Eighteen adult male Wistar rats (weighing 250–270 g) obtained from BioLASCO (Taipei, Taiwan) were randomly divided into three groups with six rats in each group. Animals in the first group were subjected to CSD (CSD group) in which CSD was performed by suffering from three cycles of 5 days of total sleep deprivation followed by 2 days of rest. During the CSD period, 2 mL of olive oil was daily administered to the CSD animals between 9:00 and 9:30 h. Animals in the second group were also subjected to CSD wherein ALA (T5625, Sigma, St. Louis, MO, USA) at the dose of 100 mg/kg dissolved in 2 mL of olive oil were given via the oral gavage every day during the entire CSD period (CSD + ALA group). Animals in the third group were housed in the same CSD apparatus but were allowed to sleep (serving as yoked control for the CSD, CSC group). During the whole experimental period, all animals were kept under the automatically regulated light–dark cycle of 12:12 h with constant room temperature of 25 ± 1 °C.

### Chronic sleep deprivation procedure

Sleep deprivation was achieved by the disk-over-water (DOW) method as described in our previous studies (21, 22). This method has been widely used and accepted for inducing SD, as it does not cause excessive physical exertion (23). Briefly, the device was composed of two clear rectangular chambers and a round plastic disk that served as a rat-carrying platform. The chambers were placed side by side and filled with water to a depth of 5 cm. The disk was built into the lower quarter of the two chambers and extended to the two chamber walls. Before the experiment, all rats were allowed to adapt to the device for 3 days. Sleep deprivation depended on the rats’ aversion to water, as rats rarely enter water spontaneously. When sleep onset was detected in a sleep-deprived rat, the disc was slowly rotated at a moderate speed of 3.5 rpm by a computerized monitoring system, forcing both rats to remain awake and walk against the direction of disc rotation to avoid being forced into the water. When a sleep-deprived rat was spontaneously awoken, the disc became stationary, and the yoked control rat had an opportunity to sleep. Electroencephalographic and electromyographic data were recorded on a Grass model 78 polygraph (Grass-Telefactor, West Warwick, RI, USA) and relayed to a computer for digital recording. All experimental procedures were approved by the Laboratory Animal Center of the Chung Shan Medical University (IACUC approval number: 2424).

### Perfusion and tissue preparation

At the end of the experimental period, half of the animals in each group were subjected to transcardial perfusion for immunohistochemical staining and spectrometric analysis. After deeply anesthetized via intraperitoneal injection of Zoletil (40 mg/kg) and Xylazine (10 mg/kg), the animals were perfused transcardially with 0.9% saline followed by 300 mL of 4% paraformaldehyde in 0.1 M phosphate buffer (PB), pH 7.4. After perfusion, the liver was removed and placed in the fixative for 2 h. Following that, the tissue samples were immersed in graded concentrations of sucrose buffer (10–30%) for cryoprotection at 4 °C overnight. Serial 30 μm-thick sections of the liver were then cut transversely with a cryostat (CM3050S, Leica Microsystems, Wetzlar, Germany) on the next day, and were alternatively placed into ninth wells of a cell culture plate. Sections collected in the first well were processed for time-of-flight secondary ion mass spectrometer (TOF-SIMS) analysis, and those in the second to eighth wells were processed for NF-κB, IL-6, cyclooxygenase-2 (COX2), B-cell lymphoma 2 (Bcl-2), Bcl-2 associated X protein (BAX), glutathione peroxidase 4 (Gpx4), and long-chain-fatty-acid-CoA ligase 4 (FACL4) immunohistochemistry, respectively. For sections collected in the last well, the terminal deoxynucleotidyl transferase dUTP nick end labeling (TUNEL) was processed to detect the potential apoptotic change.

### Measurement of serum levels of key metabolic parameters and hepatic enzymes

Blood samples collected from the left ventricle of the heart during perfusion were firstly centrifuged at 1,500×*g* for 20 min to remove debris. Supernatants were then collected and the serum levels of AST, ALT, ALP, triglyceride (TG), cholesterol (CHO), high-density lipoproteins (HDL), and low-density lipoproteins (LDL) were determined using commercially available reagents (Roche-Diagnostics, Indianapolis, IN, USA) in accordance with the standard operating procedures recommended by the manufacturers.

### Time-of-flight secondary ion mass spectrometer (TOF-SIMS) analysis

In order to evaluate the intensity and distribution of Fe^2+^ in the liver tissue, spectrometric analysis was performed by using the TOF-SIMS IV instrument (ULVAC-PHI TRIFT IV, Kanagawa, Japan). Briefly, samples collected from each group were affixed to silica wafers (1 cm × 1 cm) with the sample holder temperature setting at −60 °C. The primary ion source was the bismuth (Bi^+^) ion gun operating at 25 kV with a 1 pA pulse current. The primary ion beam was scanned over an area of 50 μm^2^, consisting of 62 × 62 pixels. Four different regions within the liver tissue were selected for scanning and four spectra were acquired from each sample. Image data acquisition time was 200 s, with charge compensation executed by a pulsed flood gun using low-energy electrons. The vacuum in the main chamber was maintained between 10^−7^ and 10^−8^ Torr, achieving a resolution of m/Δm = 7,450. For quantification, the spectral intensity detected in each section was normalized to the ionic intensity of paraformaldehyde (set as baseline = 100%) and presented as a percentage above the baseline. The normalized spectra from each sample (4 spectra per section, 30 slices per animal) were then averaged to generate representative data for that specific animal.

### Immunohistochemical staining

With the purpose to determine the spatial expression of pro-inflammatory cytokine as well as the apoptotic and ferroptotic markers, liver sections collected from each group were firstly immersed in phosphate buffer saline (PBS), pH 7.4, containing 3% H_2_O_2_/methanol solution for 1 h to reduce the endogenous peroxidase activity. Following that, the sections were incubated in blocking medium containing 0.1% Triton X-100, 3% normal goat serum, and 2% bovine serum albumin for 1 h to block nonspecific binding. Subsequently, the sections were reacted with the following primary antibodies: NF-κB (p65) (1:200, Santa Cruz, sc-8008), IL-6 (1:500, Bioss bs-0782R-TR), COX2 (1:200, Santa Cruz, sc-376861), BAX (1:100, Santa Cruz, sc-7480), Bcl-2 (1:200, Millipore, AB1722), GPx4 (1:100, Abcam, ab125066), and FACL4 (1:200, Abcam, ab155282) in blocking medium for 48 h at 4 °C. After several rinses with 0.01 M PBS, the sections were placed in secondary antibody (1:200, Vector Laboratories, Burlingame, CA, USA) at room temperature for 2 h. Subsequently, all sections were immersed in 1:100 concentrations of avidin-biotin complex for 1 h at room temperature and then in 1.5% DAB (3,3-diaminobenzidine) solution containing 0.02% H_2_O_2_ as chromogen. Finally, all reacted sections were rapidly dehydrated through a series of graded alcohols, cleared with xylene, and coverslipped with Permount.

### Western blot analysis

For biochemical analysis of protein activity, another half of the animals in each group were deeply anesthetized with intraperitoneal injection of ketamine (100 mg/kg) and xylazine (10 mg/kg) and sacrificed by cervical dislocation immediately. The liver was quickly removed and rapidly transferred to liquid nitrogen until subsequent use. The tissues collected from each group were collected in 0.01 M PBS with protease inhibitors [2 g/mL aprotinin, leupeptin, pepstatin A, and 120 g/mL phenylmethanesulfonyl fluoride (PMSF)] and homogenized (Polytron RT MR3100) at 3 × 10-s intervals. The homogenates were then spun at 13,200 rpm at 4 °C for 30 min to remove debris. The supernatant was then collected and protein was assayed on 96-well plates by adding 3 μL of standard and 150 μL of protein assay dye reagent (Bio-Rad, Hercules, CA, USA) diluted 1:5 with distilled water. Absorbance at 595 nm was measured immediately on an automated plate reader. Equal amounts of protein were subjected to SDS gel electrophoresis and transferred to nitrocellulose (NC) paper. Immunodetection was applied with primary antibodies specific to erythroid 2-related factor 2 (Nrf2) (1:5,000, Affinity, AF0639), phospho-Nrf2 (p-Nrf2) (1:1,000, Affinity, DF7519), superoxide dismutase 1 (SOD1) (1:1,000, Thermo, PA1–30195), glutathione peroxidase (GPx) (1:1,000, Abcam, Ab22604), catalase (1:2,000, Santa Cruz, sc-271803), NF-κB (p-65) (1:250, Santa Cruz, sc-8008), TNF-α (1:200, Santa Cruz, sc-52746), IL-6 (1:500, Bioss, bs-0782R-TR), IL-1β (1:100, Santa Cruz, sc-52012), COX2 (1:200, Santa Cruz, sc-376861), activating transcription factor 6α (ATF6α) (1:200, Santa Cruz, sc-166659), inositol-requiring enzyme 1α (IRE1α) (1:250, Santa Cruz, sc-390960), protein kinase R-like endoplasmic reticulum kinase (PERK) (1:100, Santa Cruz, sc-377400), caspase 3 (1:200, Santa Cruz, sc-56053), and β-actin (1:5,000, Novus, NB600-501), respectively. The immuno-signal was detected by reacting with horseradish peroxidase (HRP)-conjugated secondary antibody (1:5,000) at room temperature for 1 h. The signals were then observed by chemiluminescence (Renaissance kit; NEN, Boston, MA, USA). ImageJ image analysis software (Version 1.4, National Institutes of Health, Bethesda, MD, USA) was used to quantify the signal intensity. Equal loading of the protein was confirmed by probing the gels running parallel to β-actin. All optical density (OD) readings were normalized for β-actin and expressed as mean ± standard deviation.

### Biochemical measurement of malondialdehyde (MDA) levels

Concerning malondialdehyde (MDA) is the main product produced by lipid peroxidation, the level of MDA has been extensively used as a reliable indicator of oxidative stress. The hepatic tissues collected from each group were subjected to homogenization firstly. Following homogenization and centrifuging, the supernatants were then used for measuring the MDA by fluorescence product generated from the reaction of this aldehyde with thiobarbituric acid. The results were determined spectrophotometrically at 532 nm and expressed as nmol/mg protein.

### Terminal deoxynucleotidyl transferase dUTP nick end labeling (TUNEL)

To detect the potential DNA fragmentation in cell nuclei arose from apoptotic changes, the TUNEL reaction was applied to the sections collected in the ninth well. After several washed in 0.1 M PB, the sections were treated with 20 g/mL proteinase K for 10 min, followed by 0.3% H_2_O_2_ in methanol for 10 min and 0.1% Triton X-100 in 0.1% sodium citrate for 2 min on ice. After that, the sections were incubated with TUNEL reaction mixture (Roche, Berlin, Germany) for 60 min at 37 °C. The reaction product was stained with DAB solution for 10 min at room temperature.

### Computerized quantitative image analysis

The approach for computerized image analysis was similar to our previous studies (21, 22). A computer-assisted image analysis system, together with the Image-Pro Plus software (Media Cybernetics, Silver Spring, MD, USA), was used to quantify the staining intensities of the BAX and Bcl-2 immunohistochemistry. The hepatic sections reacted for BAX and Bcl-2 were densitometrically measured by a digital camera mounted on a ZEISS microscope (Axioplane 2, Carl Zeiss MicroImaging GmbH, Hamburg, Germany). All densitometric readings taken from each image (area of measurement = 200 μm^2^) were then combined and averaged to obtain the total optical density (TOD) of each image. The background staining (BOD) of each image was measured by calculating the optical density of the area of central vein. True OD for each image was then expressed by subtracting the BOD from TOD, so that each measurement was made in an unbiased way to correct for background. As actual level of reaction product accumulated in the tissue section as a result of immunohistochemical reactivity is influenced by lots of factors, all parameters such as gray level adjustment, histogram stretch, and minimal optical density were carefully controlled following the recommended procedures.

### Statistical analysis

SPSS 22.0 version statistical software (IBM Corporation, Armank, NY, USA) was used for statistical analysis. One-way analysis of variance (ANOVA) followed by Bonferroni *post hoc* test were applied for determination of significance. Statistical significance was defined as *p* < 0.05.

## Results

### ALA successfully improves metabolic function following CSD

The serum levels of AST, ALT, ALP, triglyceride, cholesterol, HDL, and LDL were assessed as biochemical indicators of metabolic function (24). The results showed that a significant up-regulation of AST, ALT, ALP, and cholesterol concentration was detected in the serum of animals subjected to CSD, suggesting that CSD significantly impaired the metabolic activity (Table 1). However, in animals subjected to CSD and treated with ALA, the serum levels of above four parameters were significantly returned to the levels comparable to those of the control ones (Table 1). This result indicated that ALA supplementation successfully ameliorates the metabolic dysfunction following CSD, potentially by preserving the hepatic metabolic enzymes, and regulating the lipid components involved in energy transport and storage.

**Table 1:** The serum levels of some biochemical markers in CSC, CSD, and CSD + ALA groups.

Groups	CSC	CSD	CSD + ALA
AST (U/L)	94.7 ± 10.07	165.6 ± 1.25^*^	133.3 ± 17.32^*#^
ALT (U/L)	33.6 ± 1.69	59.3 ± 5.9^*^	47 ± 4.96^*#^
ALP (U/L)	367.66 ± 17.91	442.34 ± 15.62^*^	294.6 ± 6.34^*#^
HDL-c (mg/dL)	22.7 ± 3.09	21.33 ± 2.05	23.3 ± 0.47
LDL-c (mg/dL)	38.67 ± 5.9	46.67 ± 7.84	37.4 ± 0.94
Cholesterol (mg/dL)	60 ± 0.81	76.4 ± 8.37^*^	49.3 ± 11.58^#^
TG (mg/dL)	45.4 ± 6.65	42.7 ± 5.9	21.7 ± 4.1^*#^

Data are presented as means ± SEM and evaluated by ANOVA. ^*^p < 0.05 vs. CSC group; ^#^*p* < 0.05 vs. CSD group.

### ALA effectively stabilizes hepatic redox-active iron (Fe^2+^) expression and depresses the formation of ferroptosis after CSD

**Figure 1:**
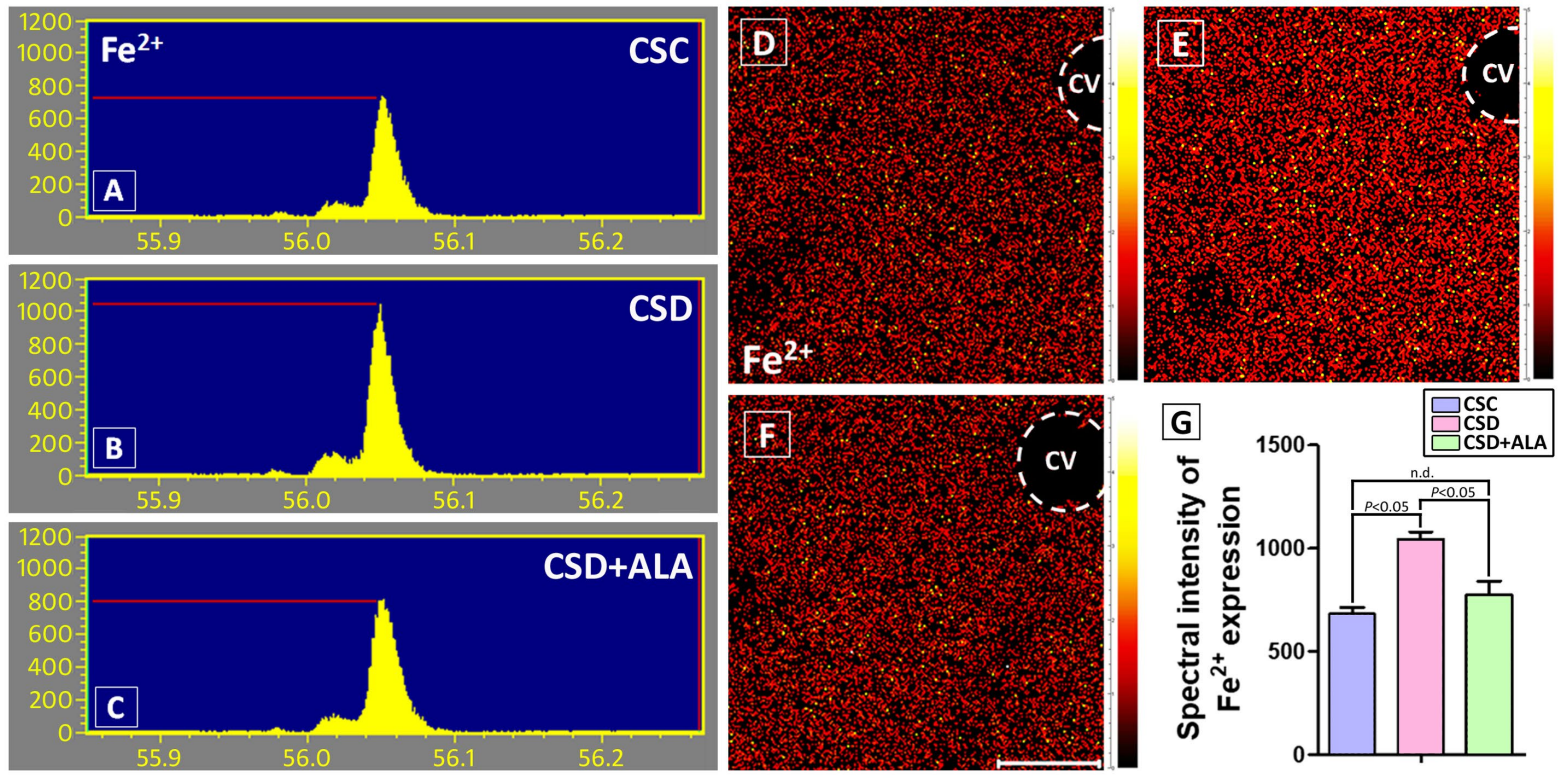
TOF-SIMS positive spectra **(A–C)**, ionic imaging **(D–F)**, and histogram **(G)** showed the Fe^2+^ expression in the liver of control (CSC), chronic sleep deprivation (CSD), and chronic sleep deprivation with alpha lipoic acid treatment (CSD + ALA) rats. The ionic imaging of Fe^2+^ expression is expressed by a color scale in which bright colors represent high levels of Fe^2+^ signaling. Note that in the control group, lesser Fe^2+^ intensity was detected in the liver tissue **(A,D)**. However, following CSD, the hepatic Fe^2+^ expression was drastically increased in both terms of spectral intensity **(B)** and ionic imaging **(E)**. Nevertheless, in CSD rats received ALA treatment, a remarkable decrease of Fe^2+^ expression was detected in the liver wherein the degree of Fe^2+^ expression was effectively returned to near the level of control ones **(C,F)**. Quantitative data from spectral intensity analysis corresponded well with ionic imaging in which ALA significantly depressed hepatic Fe^2+^ intensity in rats suffered from CSD **(G)**. These data clearly indicate that ALA treatment effectively prevent hepatic Fe^2+^ overload induced by CSD, and thereby, counteracting the CSD-induced metabolic deficiency. CV: Central Vein. Scale bar = 100 μm.

**Figure 2:**
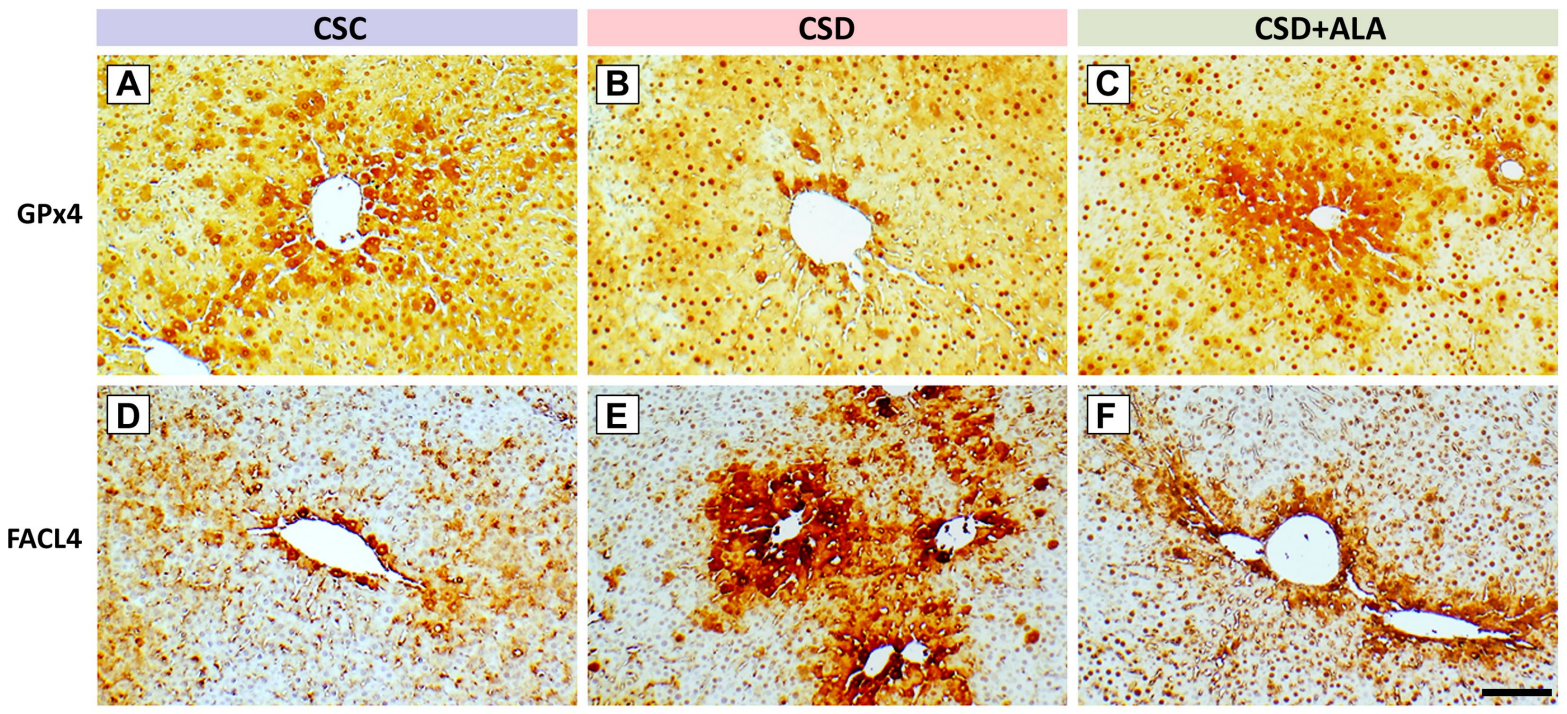
Photomicrographs **(A–F)** showed the immunohistochemical staining of glutathione peroxidase 4 (GPx4) **(A–C)** and long-chain-fatty-acid-CoA ligase 4 (FACL4) **(D–F)** in the liver of control (CSC), chronic sleep deprivation (CSD), and chronic sleep deprivation with alpha lipoic acid treatment (CSD + ALA) rats. Note that CSD significantly reduced the immuno-expression of GPx4 **(B)**, a key inhibitor of ferroptosis, while markedly upregulating FACL4 **(E)**, a protein that sensitizes cells to ferroptotic death. However, in rats subjected to CSD and received ALA treatment, the expression of GPx4 was considerably increased **(C)** whereas the staining intensity of FACL4 was noticeably decreased **(F)**. These results clearly indicate that ALA treatment successfully suppresses hepatic ferroptosis by restoring the balance between GPx4–ACSL4 axis that prevents cellular susceptibility to the development of ferroptosis. CV: Central Vein. Scale bar = 100 μm.

Considering that overload of the redox-active iron (Fe^2+^) plays a harmful role in hepatic metabolic function due to its involvement in catalyzing the Fenton reaction, generating ROS, enhancing oxidative stress, exacerbating inflammatory signaling, and leading to the development of ferroptosis, the present study further examined the hepatic Fe^2+^ expression, together with the ferroptotic related proteins by the use of TOF-SIMS analysis and immunohistochemistry, respectively. Data from spectrometric analysis revealed that the ion intensity of Fe^2+^ in the control group was estimated to be 681.6 ± 44.7 (Figures 1A,G). However, following CSD, the intensity of the hepatic Fe^2+^ was drastically increased to 1043.3 ± 51.6 (Figures 1B,G). Results from ionic image corresponded well with the spectrometric finding in which noticeably overload of the hepatic Fe^2+^ expression was detected following CSD (Figure 1E) as compared to that of the control one (Figure 1D). Concomitant with the spectrometric data, the immuno-expression of GPx4, a key inhibitor of ferroptosis, was significantly decreased (Figure 2B) while the staining level of FACL4, a protein that promotes ferroptosis by enhancing lipid peroxidation, was remarkably increased following CSD (Figure 2E). Nevertheless, in animals subjected to CSD and treated with ALA, the Fe^2+^ intensity was significantly decreased to 775 ± 93.54 (Figures 1C,G). Comparable to these findings, the immuno-reactivities of both GPx4 and FACL4 were successfully preserved to near the normal levels under ALA treatment during CSD (Figures 2C,F). These results indicated that CSD would impair the hepatic redox homeostasis and contribute to the development of ferroptosis, which could effectively be rescued by supplementation with ALA.

### ALA remarkably reduces oxidative stress by preserving Nrf2 and its downstream anti-oxidative enzyme activity

**Figure 3:**
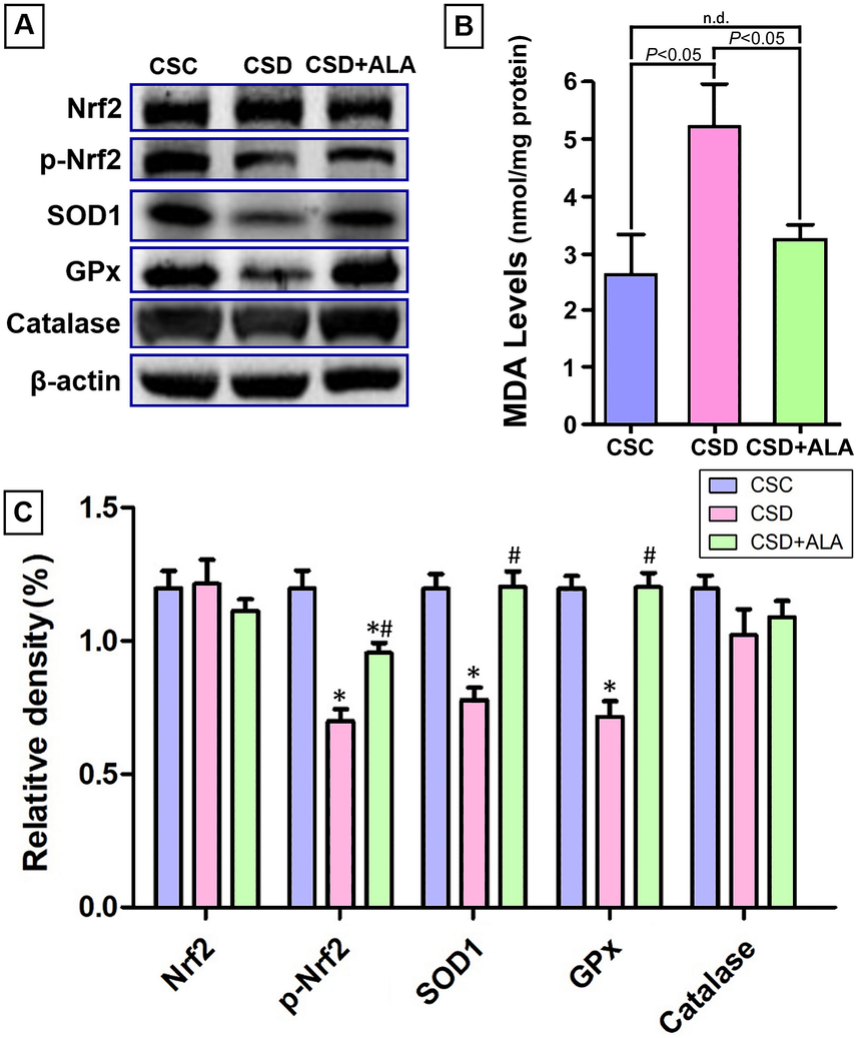
Immunoblot **(A)** and histograms **(B,C)** showed the total Nrf2, phosphorylated Nrf2 (p-Nrf2), main downstream anti-oxidative enzymes expressions [i.e. superoxide dismutase 1 (SOD1), glutathione peroxidase (GPx), and catalase] and the malondialdehyde (MDA) level **(B)** in the liver of control (CSC), chronic sleep deprivation (CSD), and chronic sleep deprivation with alpha lipoic acid treatment (CSD + ALA) rats. Note that the activities of hepatic p-Nrf2, SOD1, and GPx were significantly decreased following CSD. However, in rats subjected to CSD and received ALA treatment, the hepatic p-Nrf2, SOD1, and GPx activities were effectively preserved to near the control levels **(A,C)**. The degree of oxidative stress paralleled well with the immunoblot findings in which the level of MDA was markedly reduced in rats receiving ADA treatment during CSD. These results clearly indicate that ALA treatment successfully restore Nrf2-mediated anti-oxidative enzyme expression, and protect liver from CSD-induced oxidative injury. *p < 0.05 compared to control value; ^#^*p* < 0.05 compared to CSD group.

The anti-oxidative action of ALA was demonstrated by evaluating its effects on MDA level, the activation of Nrf2, and Nrf2-mediated downstream anti-oxidative enzymes’ activities. The immunoblot results revealed that CSD significantly depressed the phosphorylated Nrf2 (p-Nrf2) activation, and subsequently reduced the downstream anti-oxidative enzymes’ activities (i.e., SOD1 and GPx) (Figures 3A,C). However, in animals suffered from CSD and received ALA treatment, the activities of both p-Nrf2 and its downstream anti-oxidative enzymes were considerably increased to near the control levels (Figures 3A,C). In a good agreement with the immunoblot data, the MDA level was markedly increased following CSD (Figure 3B). The increment of MDA induced by CSD was effectively reduced by ALA treatment (Figure 3B). Although the activity of catalase did not affect by the CSD and ALA, the present study clearly demonstrates that ALA successfully exerts its hepatoprotective function by remarkably restoring the p-Nrf2-mediated downstream anti-oxidative enzymes’ activation, and therefore consequently reduce the degree of oxidative stress.

### ALA considerably suppresses CSD-induced inflammatory reaction

**Figure 4:**
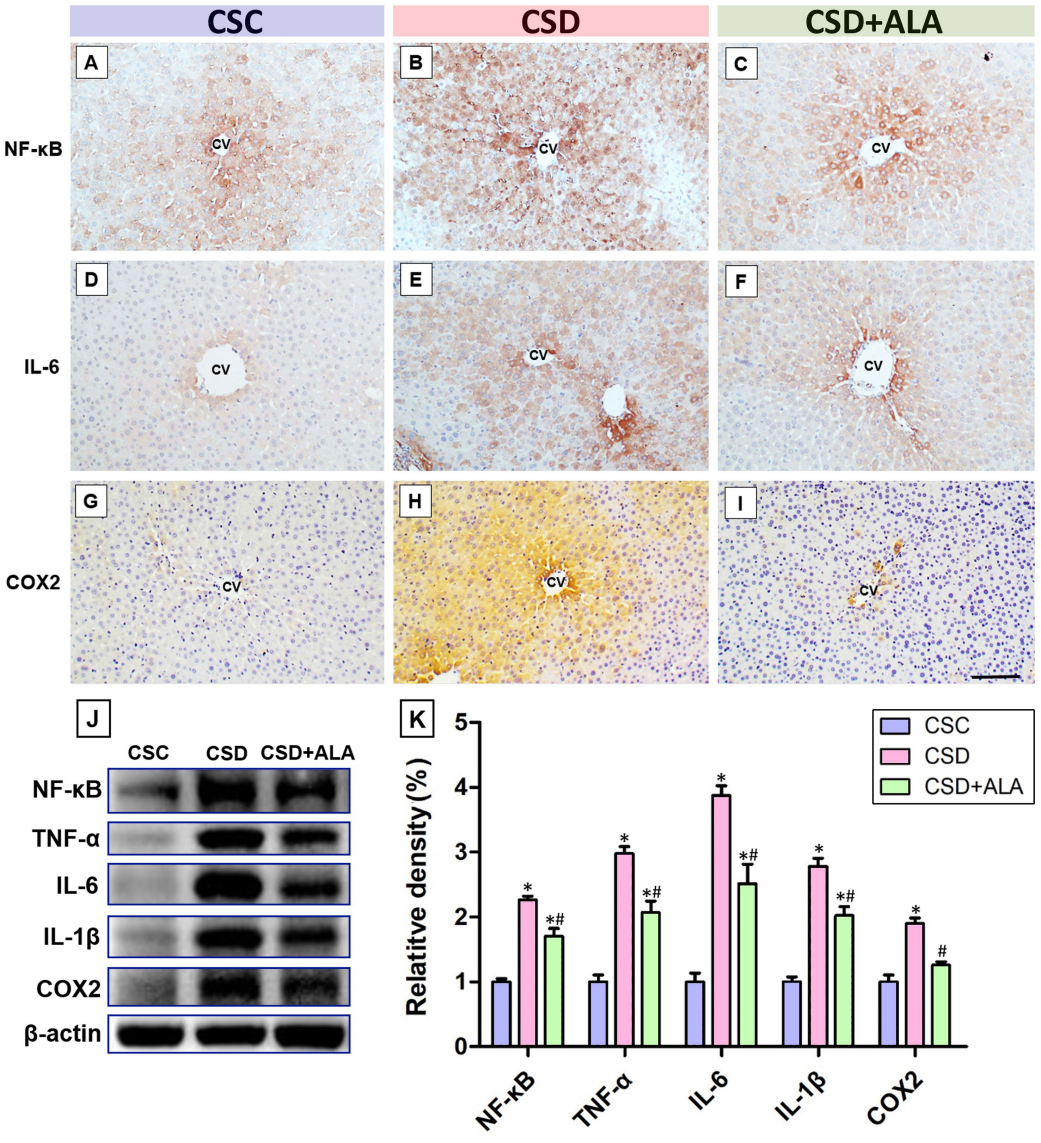
Photomicrographs **(A–I)**, immunoblot **(J)**, and histogram **(K)** showed the expression of key inflammatory factors in the liver of control (CSC), chronic sleep deprivation (CSD), and chronic sleep deprivation with alpha lipoic acid treatment (CSD + ALA) rats. Note that the expressions of all the detected inflammatory factors (such as NF-κB, TNF-α, IL-6, IL-1β, and COX2) were remarkably increased in the liver following CSD as demonstrated by both immunohistochemical staining **(B,E,H)** and Western blotting **(J)**. Also note that in rats subjected to CSD and received ALA treatment, the expression of all above inflammatory factors were significantly decreased to near the levels of the control ones **(C,F,I,J,K)**. These results clearly indicate that CSD would considerably induce hepatic inflammation, which could successfully be depressed by ALA treatment. *p < 0.05 compared to control value; ^#^*p* < 0.05 compared to CSD group. CV: Central Vein. Scale bar = 100 μm.

As shown in Figure 4, CSD significantly induced hepatic inflammation by noticeably up-regulating NF-κB-mediated inflammatory reaction, and increased the production of several key inflammatory factors such TNF-α, IL-1β, IL-6, and COX2 in hepatic tissues as examined by both immunohistochemical (Figures 4B,E,H) and biochemical approaches (Figures 4J,K). However, in animals received ALA administration during the CSD period, all above inflammatory factors were significantly decreased to the levels of the control ones (Figures 4C,F,I–K). These results clearly demonstrated that supplementation with ALA could considerably suppress the hepatic inflammation resulted from CSD.

### ALA significantly inhibits the expression of signaling proteins of ER stress following CSD

**Figure 5:**
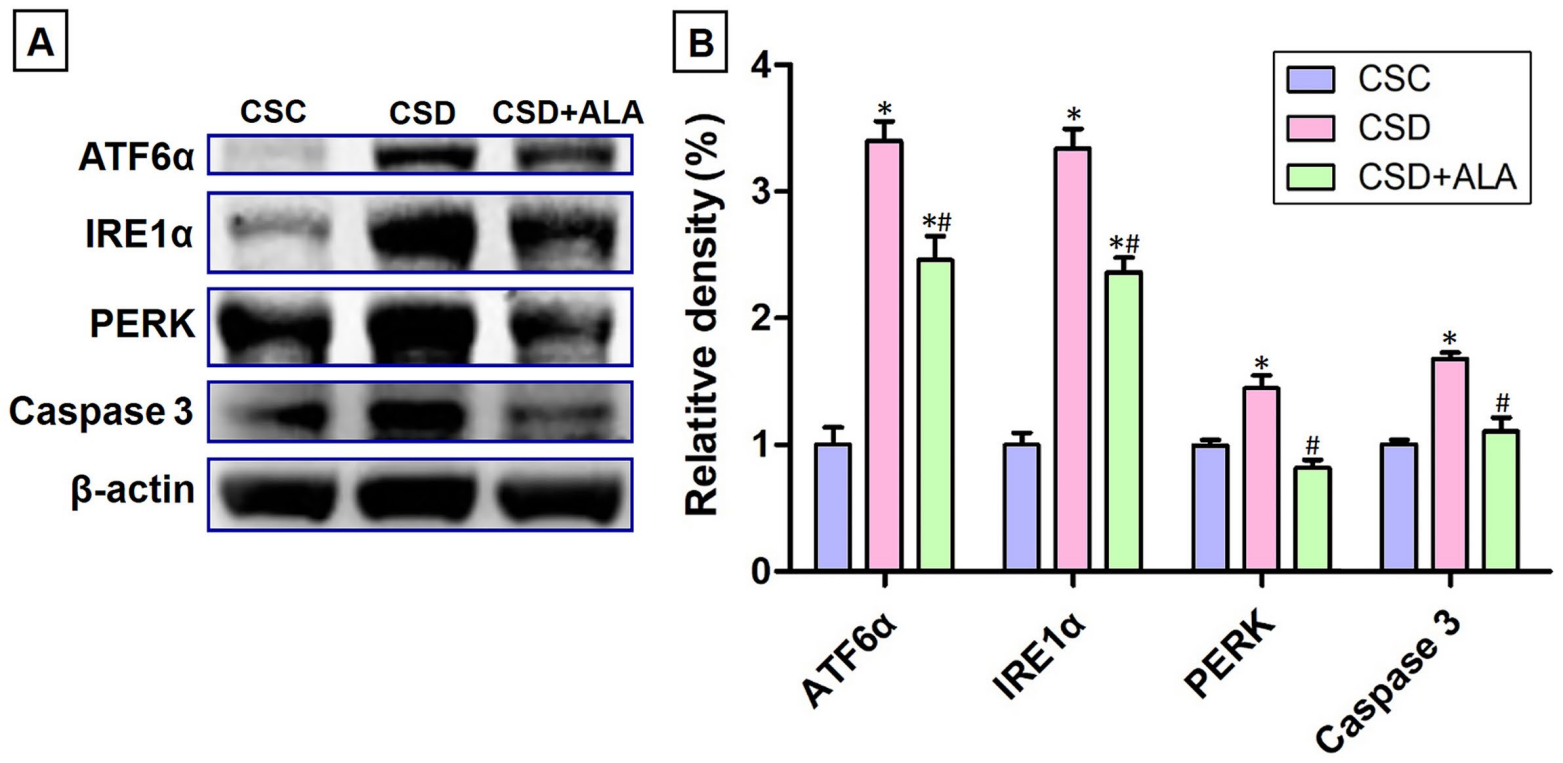
Immunoblot **(A)** and histogram **(B)** showed the expression of signaling proteins involved in ER stress in the liver of control (CSC), chronic sleep deprivation (CSD), and chronic sleep deprivation with alpha lipoic acid treatment (CSD + ALA) rats. Note that CSD remarkably increased the activities of principal ER stress proteins including ATF6α, IRE1α, and PERK. Increased activation of these proteins subsequently up-regulated the expression of caspase 3, a key mediator for the initiation of apoptosis. However, in rats subjected to CSD and received ALA treatment, both the expression of ER stress proteins and caspase 3 were effectively reduced. These results clearly indicate that supplementation with ALA during CSD period would successfully inhibit ER stress-mediated apoptosis, which may therefore contribute to the maintenance of healthy metabolic activity. *p < 0.05 compared to control value; ^#^*p* < 0.05 compared to CSD group.

In order to further investigate whether ALA would exert the hepatoprotective effects through modulating the activities of endoplasmic reticulum stress (ER stress) following CSD, the expressions of signaling proteins involved in the process of ER stress (such as ATF6α, IRE1α, and PERK) were quantitatively examined by the immunoblotting analysis. Biochemical results indicated that the protein levels of ATF6α, IRE1α, and PERK were drastically increased following CSD (Figure 5A). Elevation of these proteins implies that CSD would trigger ER stress by inducing the unfolded protein response. Nevertheless, in animals subjected to CSD and received ALA administration, the levels of all above signaling proteins were noticeably reduced to near the control values (Figure 5A). Moreover, ALA administration also significantly diminished the activity of caspase 3, a crucial mediator of activating the processes of programmed cell death (Figure 5B). These findings clearly show that ALA could significantly inhibit the ER stress induced by CSD, and subsequently decreases the development of metabolic dysfunction through diminishing the caspase 3-mediated apoptotic signaling.

### ALA substantially amends the CSD-induced apoptosis and apoptotic effector protein activities

**Figure 6:**
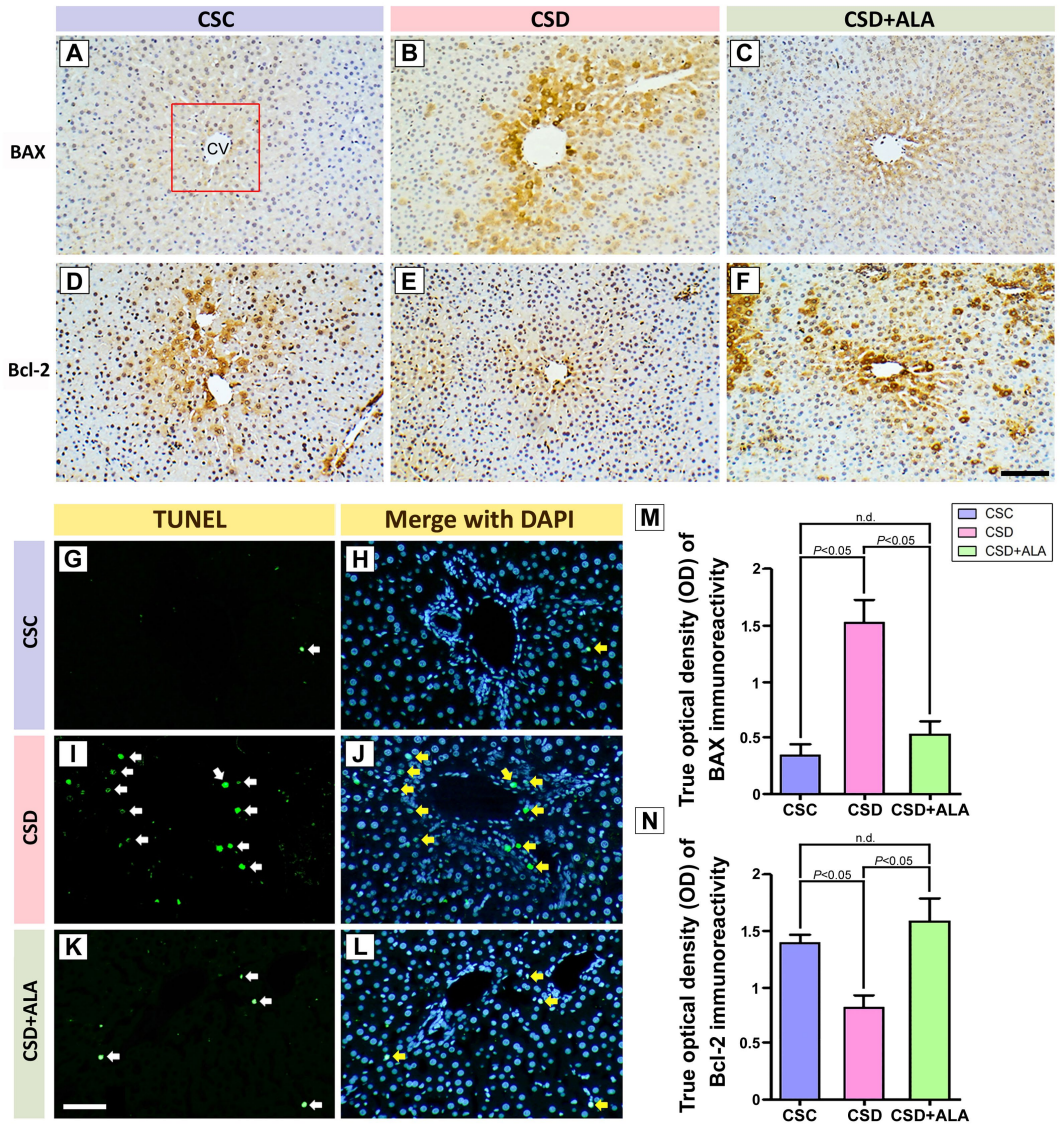
Photomicrographs **(A–L)** and histograms **(M,N)** showed the immunohistochemical staining of Bcl-2–associated X protein (BAX) **(A–C)**, B-cell lymphoma 2 (Bcl-2) **(D–F)**, TUNEL staining **(G,I,K)**, TUNEL staining merges with DAPI **(H,J,L)**, and quantitative image analysis of BAX **(M)** and Bcl-2 **(N)** staining in the liver of control (CSC), chronic sleep deprivation (CSD), and chronic sleep deprivation with alpha lipoic acid treatment (CSD + ALA) rats. Note that CSD significantly induced hepatic apoptosis [as shown by TUNEL staining **(I,J)**] through effectively promoting the activation of BAX **(B)** while simultaneously suppressing the expression of Bcl-2 **(E)**. However, in rats subjected to CSD and received ALA treatment, the number of apoptotic cells was markedly decreased **(K,L)**. Results obtained from TUNEL staining coincided well with immunohistochemical findings in which the expression of BAX was considerably decreased **(C)** whereas the staining level of Bcl-2 was noticeably increased **(F)** following ALA supplementation. Quantitative data confirmed the immunohistochemical evidence that ALA treatment successfully restored the staining intensity of BAX and Bcl-2 to normal levels **(M,N)**. These results clearly indicate that ALA treatment successfully suppresses hepatic apoptosis by restoring the balance between pre- and anti-apoptosis proteins, thereby preserving the metabolic function against CSD-induced injury. The red square indicates the area used for quantitative image analysis of each section. CV: Central Vein. Scale bar = 100 μm.

As attempts to further elucidate the potential effects of ALA on apoptosis and the main apoptotic effector protein activities, the level of apoptosis and the expression pattern of pro-apoptotic protein (BAX)/anti-apoptotic protein (Bcl-2) were morphologically detected by TUNEL and quantitative immunohistochemistry, respectively. Data collected from immunohistochemical staining revealed that significant up-regulation of BAX expression as well as remarkable down-regulation of Bcl-2 staining were observed in hepatic tissues following CSD (Figures 6B,E,M,N). The increased production of the BAX protein coincided well with the higher expression of caspase 3 after CSD (Figure 5), which plays a crucial role in apoptosis by promoting the release of cytochrome c and triggering the caspase 3-mediated apoptotic signaling. However, in animals received ALA treatment during the CSD period, the hepatic BAX expression was effectively decreased (Figures 6C,M). Paralleled well with the reduction of BAX expression, ALA treatment also significantly restored the protein level of Bcl-2 in which the hepatic Bcl-2 staining was successfully preserved in ALA-treated rats following CSD (Figures 6F,N). Results obtained from TUNEL also coincided well with the immunohistochemical findings wherein ALA treatment successfully decreased the number of apoptotic cells induced by CSD (Figures 6J,L). These findings support the anti-apoptotic effects of ALA after CSD, mainly through amending the apoptotic effector proteins’ activities.

## Discussion

The present study provides the compelling evidence that ALA treatment successfully improves metabolic function following CSD, primarily through its potent anti-oxidative, anti-inflammatory, and anti-apoptotic activities. By employing a combination of spectrometric, biochemical, and morphological approaches, this study clearly demonstrated that ALA effectively stabilizes hepatic Fe^2+^ expression (Figure 1), alleviates ferroptotic changes (Figure 2), reduces oxidative stress by enhancing phosphorylated Nrf-2 activation-mediated downstream anti-oxidative enzymes’ expressions (Figure 3), decreases the production of NF-κB-mediated inflammatory factors (Figure 4), depresses the activation of ER stress signaling (Figure 5), as well as counteracts the imbalance of pro- and anti-apoptotic proteins following CSD (Figure 6). All of these effects collectively contribute to the improvement of hepatic function and metabolic activity (Table 1). It is well known that CSD is close associated with increased risk of metabolic dysfunction (such as obesity, diabetes, and high blood pressure), which has become the most common problem in our modern society (1–3). Enhanced oxidative stress and extensive inflammatory reaction has long been reported to serve as the underlying mechanisms for the pathogenesis of CSD-induced metabolic deficiency (8, 9). Through DNA damage, lipid peroxidation, protein oxidation, and the release of pro-inflammatory factors, CSD would disrupt hepatic cellular membrane integrity and alter the structural proteins of iron channels, leading to Fe^2+^ overload and exacerbating the oxidative stress-mediated ferroptotic changes (8, 25, 26). This is the case that in our current study, we clearly detected a significant increase of Fe^2+^ signal (Figure 1), and noticeable changes in the balance of GPx4 and FACL4 following CSD (Figure 2). As GPx4 and FACL4 act as opposing regulators of ferroptosis, disrupted GPx4 and FACL4 expression would therefore promote the susceptibility of hepatocytes to ferroptotic cell death (27). Moreover, excessive Fe^2+^ overload would further feed back into apoptosis by inducing ER stress and activating pro-apoptotic pathway (28), which ultimately worsen metabolic dysfunction and liver injury. Results from the present study further corresponded well with this viewpoint in which increment of Fe^2+^ expression coincided well with the up-regulation of ATF6α, IRE1α, PERK, and BAX activities (Figures 5, 6), all are key elements participated in the development of ER stress and apoptosis. Considering that ALA could effectively stabilize hepatic Fe^2+^ expression (Figure 1), and depress both levels of ferroptotic (Figure 2) and pro-apoptotic signaling (Figure 6), dietary supplementation with ALA may thus serve as a useful and practical way to prevent, or further counteract the CSD-induced liver injury and metabolic deficiency.

In addition to improve the metabolic function through directly exerting the anti-oxidative and anti-inflammatory effects, ALA also plays an important role in energy metabolism through its involvement in mitochondrial multi-enzyme complexes (19, 29). It has been reported that ALA could preserve the structural integrity of cytochrome c, and prevent cytochrome c release from mitochondria during metabolically or oxidatively challenging conditions (18, 30). This process is essential for reducing the initiation of intrinsic apoptotic pathways, which ultimately contributes to the enhancement of mitochondrial ATP production and overall metabolic activity (31, 32). In a good agreement with this suggestion, the present study further demonstrated that ALA treatment successfully suppressed the increased levels of AST, ALT, and ALP following CSD (Table 1). Reduced expression of AST, ALT, and ALP was positively correlated with the diminished release of cytochrome c that consequently inhibit the BAX and caspase 3-mediated apoptotic signaling (Figures 5, 6). Besides to modulate the mitochondrial redox status, ALA has further been reported to be implicated in the lipid regulation through its critical roles in modulating several key enzymes such as hydroxymethylglutaryl-CoA reductase (HMG-CoA reductase) and lipoprotein lipase (LPL) (33). Previous studies have indicated that ALA could suppress the expression and activity of HMG-CoA reductase, the rate-limiting enzyme responsible for *de novo* cholesterol synthesis in the liver, therefore reducing endogenous cholesterol biosynthesis (33, 34). Biochemical reports also demonstrated that ALA could enhance the activity of LPL, a key enzyme involved in hydrolyzing triglycerides in circulating chylomicrons, hence facilitating the clearance of triglycerides from the blood vessels (33–36). This is also the case that in our present study, we clearly detected the reduced levels of cholesterol and triglycerides in the serum of CSD rats treated with ALA (Table 1). As ALA could significantly suppress lipid biosynthesis and enhance lipid clearance, the protective effects of ALA on metabolic function may benefit not only from inhibiting hepatocyte apoptosis induced by mitochondrial dysfunction, but also from ALA’s regulatory effects on improving lipid profiles (34, 37).

On the other hand, it is worthy to note that although ALA treatment could effectively increase the Nrf2-mediated anti-oxidative enzymes’ activities, the increment level was not uniformly detected across all enzymes. In the present study, both SOD1 and GPx expressions were significantly upregulated in the animals subjected to CSD and treated with ALA (Figure 3). However, in animals received the same treatment, the expression of catalase was just exhibited a mild increasing trend but did not reach statistical significance as compared to that of CSD (Figure 3). Although the precise mechanisms underlying the differential expression of these anti-oxidative enzymes remain incompletely understood, one possible explanation is that their distinct affinities for hydrogen peroxide (H_2_O_2_) may play an essential role in contributing to this discrepancy. It has been reported that GPx possesses a higher affinity for H_2_O_2_ compared to catalase in isolated hepatocytes (38). From this perspective, the excessive generation of H_2_O_2_ induced by CSD in the liver may be preferentially scavenged by GPx, thereby limiting the availability of H_2_O_2_ for catalase engagement. As a result, the residual H_2_O_2_ levels may not be sufficient to significantly modulate catalase expression, which could explain the unchanged catalase levels observed following ALA treatment. Alternatively, the multifaceted role of ALA in maintaining cellular redox balance through its interaction with the glutathione (GSH) system should not be overlooked (39). It is indicated that ALA could enhance the intracellular synthesis of GSH by increasing the availability of its precursor cysteine (40). Elevated levels of GSH are critical for the optimal function of GPx that utilizes GSH as a substrate to neutralize H_2_O_2_ and lipid hydroperoxides (41). In this context, supplementation with ALA would undoubtedly support GPx activity by maintaining sufficient intracellular GSH pools, thereby leading to the significant enhancement of GPx expression following CSD as compared to other relatively unchanged anti-oxidative enzymes.

Another important issue in exploring the beneficial effects of ALA is the vehicle, dosage, and timeframe employed in the present study. In this study, we used olive oil, an agent well-known for its intrinsic anti-oxidative and hepatoprotective properties, as the vehicle for ALA. The rationale for using olive oil as the vehicle for ALA is primarily because of its high oxidative stability, which helps preserve the chemical integrity of ALA during administration. In addition, olive oil is also commonly used as a carrier for numerous lipid-soluble compounds due to its excellent compatibility with biological membranes and ease of digestion, making it an efficient medium for delivery. In the present study, equal amounts of olive oil were administered to both CSD group and CSD + ALA group. Therefore, the observed protective effects can be attributed to ALA itself, rather than to any confounding influence of olive oil, since both groups received the same level of the vehicle. As for the dosage, ALA was administered daily at a dose of 100 mg/kg. The selection for employing this dose was based on two practical considerations. First, this dose has been widely used in a variety of rat models targeting for the treatment of various disorders derived from nearly all physiological systems (42–48). Second, the current dose was approximately equivalent to the commercially available dose provided for humans (around 600 mg per capsule found in over-the-counter ALA supplements) (49). On the other hand, with regard to the timeframe, the experimental animals were received ALA treatment only during the CSD period rather than continuously afterward. This design was intended to specifically evaluate the *protective* effect of ALA against CSD-induced oxidative and inflammatory injury, rather than its *potential* role during recovery. Moreover, chronic or prolonged supplementation with ALA after the cessation of stress stimuli may not provide additional metabolic or anti-oxidative benefits, and in some cases, may partially associated with histopathological changes in liver or induce pro-oxidant effects under normal physiological conditions (50–52). Nevertheless, future studies using different doses of ALA, or continuous ALA administration throughout the post-CSD period are warranted to further enhance the translational value of the beneficial effects of ALA on counteracting the CSD-induced metabolic deficiency.

## Conclusion

**Figure 7:**
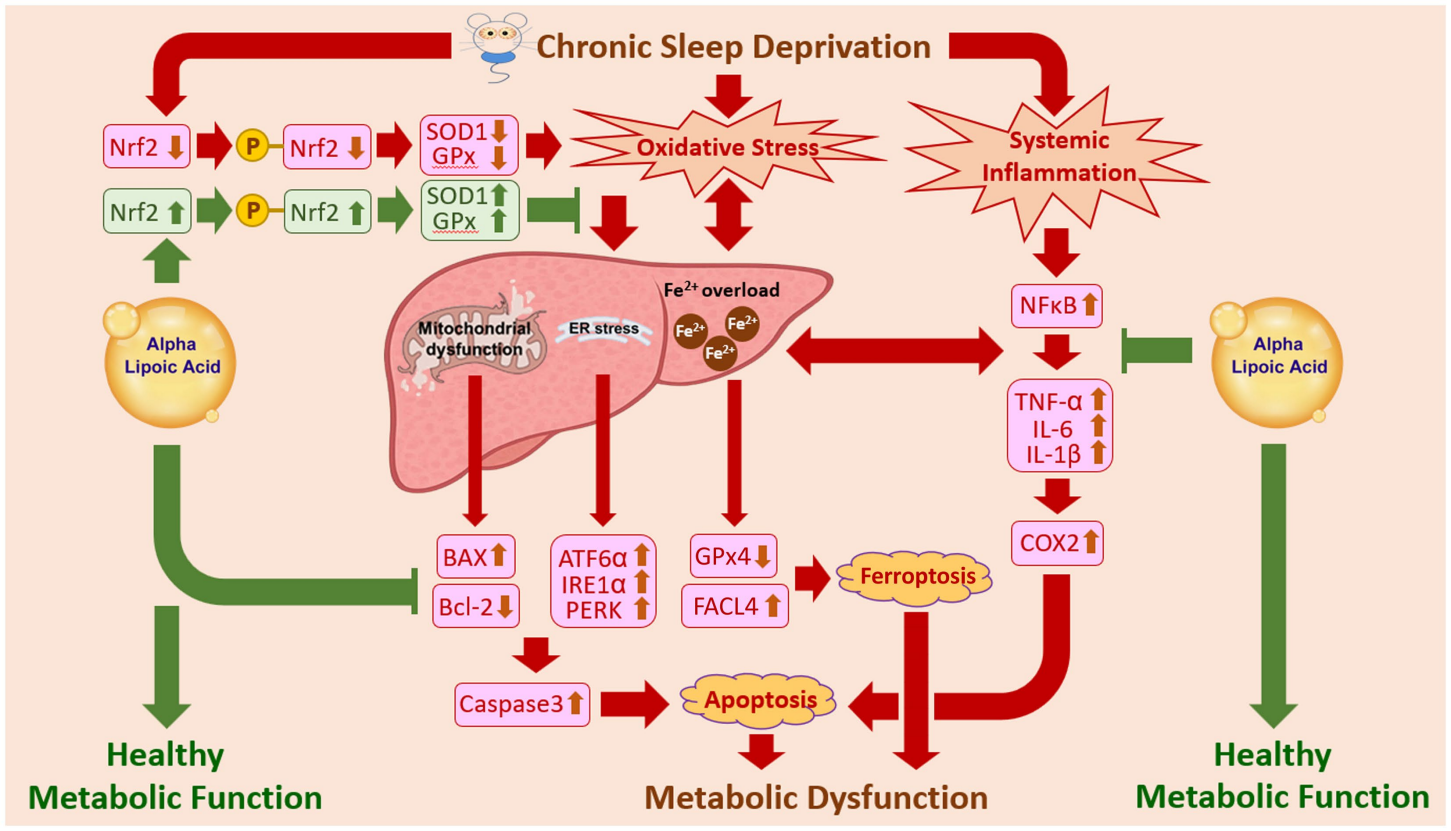
Schematic illustration of the hepatoprotective mechanisms of alpha-lipoic acid (ALA) against chronic sleep deprivation (CSD)-induced metabolic deficiency. It is demonstrated that CSD would trigger oxidative stress and systemic inflammation by reducing the anti-oxidative enzyme expression and enhancing the production of NF-κB-mediated inflammatory cytokines. These pathological changes would lead to hepatic redox-active Fe^2+^ overload, which contributes to the development of ferroptosis (by disrupting the GPx4-FACL4 axis), mitochondrial dysfunction, and ER stress in hepatocytes. Severe mitochondrial dysfunction and ER stress would then activate caspase 3-dependent apoptotic signaling pathways, including BAX/Bcl-2 imbalance and upregulation of ER stress markers (such as ATF6α, IRE1α, PERK), ultimately resulting in hepatic apoptosis and metabolic deficiency. Exogenous supplementation with ALA during CSD period successfully counteracts these pathological changes through promoting phosphorylated Nrf2 activation, restoring anti-oxidative defenses, reducing Fe^2+^ accumulation, suppressing inflammatory mediators, and effectively inhibiting ferroptotic and apoptotic signaling. As a result, these actions contribute to the improvement or protection of liver function that consequently preserve metabolic activity following CSD-induced injury.

This study is the first one to clearly demonstrate that exogenous supplementation with ALA could successfully improve the liver function and metabolic activity following chronic sleep deprivation induced injury. Through effectively attenuating the redox-active Fe^2+^ accumulation, decreasing the ferroptotic changes, enhancing the Nrf2-mediated anti-oxidative enzymes’ expression, reducing the production of key inflammatory factors, and suppressing the caspase 3-dependent apoptotic signaling, ALA remarkably protect the liver function and prevent CSD-induced metabolic deficiency (Figure 7). Although the precise mechanisms underlying the beneficial effects of ALA has not yet been fully understood, by successfully clarifying the manifold hepatoprotective function of ALA, this study sheds an important light for potential use of ALA as a dietary supplement to prevent, or even to counteract, the metabolic dysfunction induced by chronic sleep deprivation, a condition increasingly prevalent in our modern society.

## Data Availability

The original contributions presented in the study are included in the article/Supplementary material, further inquiries can be directed to the corresponding author.
