# Supplementary material for: Manifold hepatoprotective actions of α-lipoic acid on metabolic function through redox regulation, inflammatory modulation, and anti-apoptosis after chronic sleep-deprived injury
Source: Front Nutr. 2026 Jan 22;12:1679494. doi: 10.3389/fnut.2025.1679494 (PMC12872533; doi:10.3389/fnut.2025.1679494)
Supplement: Supplementary file 1 [file Image_1.pdf]

Manifold Hepatoprotective Actions of α-Lipoic Acid on Metabolic Function through Redox Regulation, Inflammatory Modulation, and Anti-apoptosis after Chronic Sleep-deprived Injury

HUNG-MING CHANG[1,2#], HSING-CHUN LIN[3,4#], TING-YI RENN[1], YU-CHENG LIU[5]
KAI-JUNG YEN[6], CHIH-KAI LIAO[6], MARIA A TIKHONOVA[7],
TAMARA G AMSTISLAVSKAYA[7], SANDEEP KUMAR SINGH[8], LI-YOU CHEN[6,9]*

Supplementary Data

Uncropped western blot images for all proteins examined.

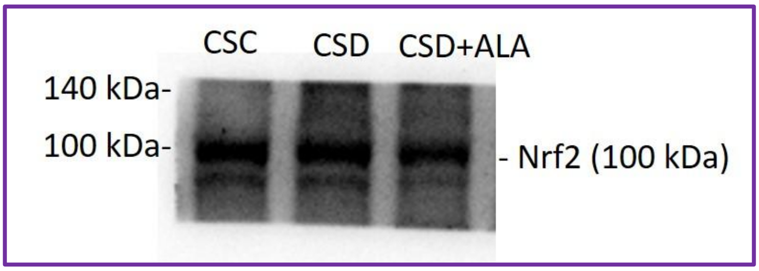

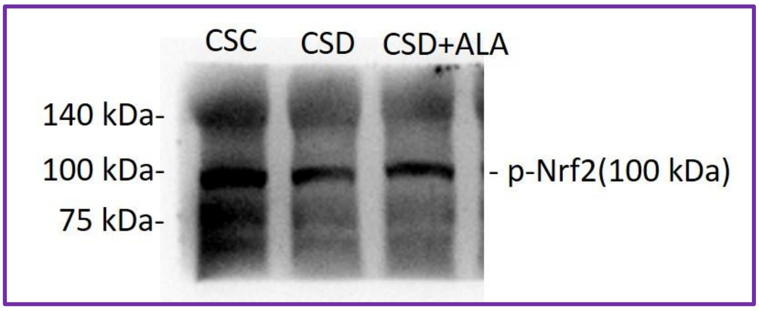

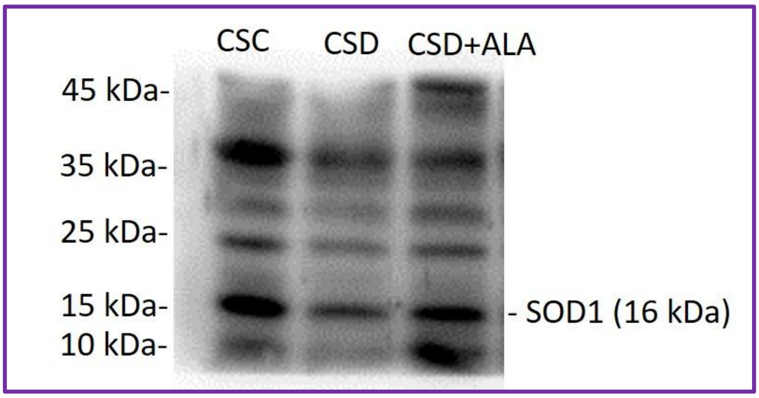

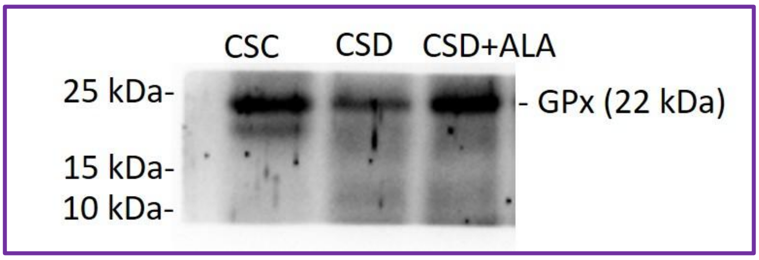

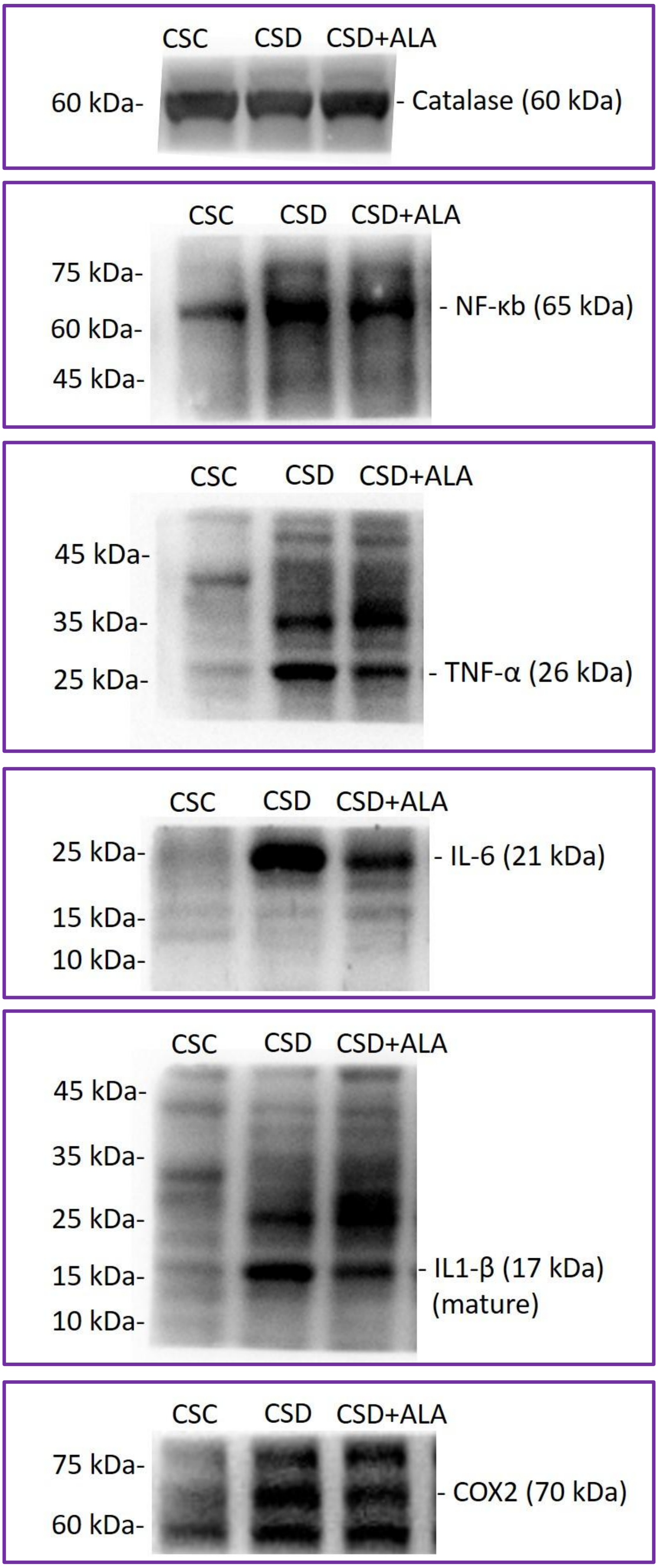
CSC
CSD
CSD+ALA
60 kDa-
- Catalase (60 kDa)
CSC
CSD
CSD+ALA
75 kDa-
60 kDa-
45 kDa-
- NF-κb (65 kDa)
CSC
CSD
CSD+ALA
45 kDa-
35 kDa-
25 kDa-
- TNF-α (26 kDa)
CSC
CSD
CSD+ALA
25 kDa-
15 kDa-
10 kDa-
- IL-6 (21 kDa)
CSC
CSD
CSD+ALA
45 kDa-
35 kDa-
25 kDa-
15 kDa-
10 kDa-
- IL1-β (17 kDa)
(mature)
CSC
CSD
CSD+ALA
75 kDa-
60 kDa-
- COX2 (70 kDa)

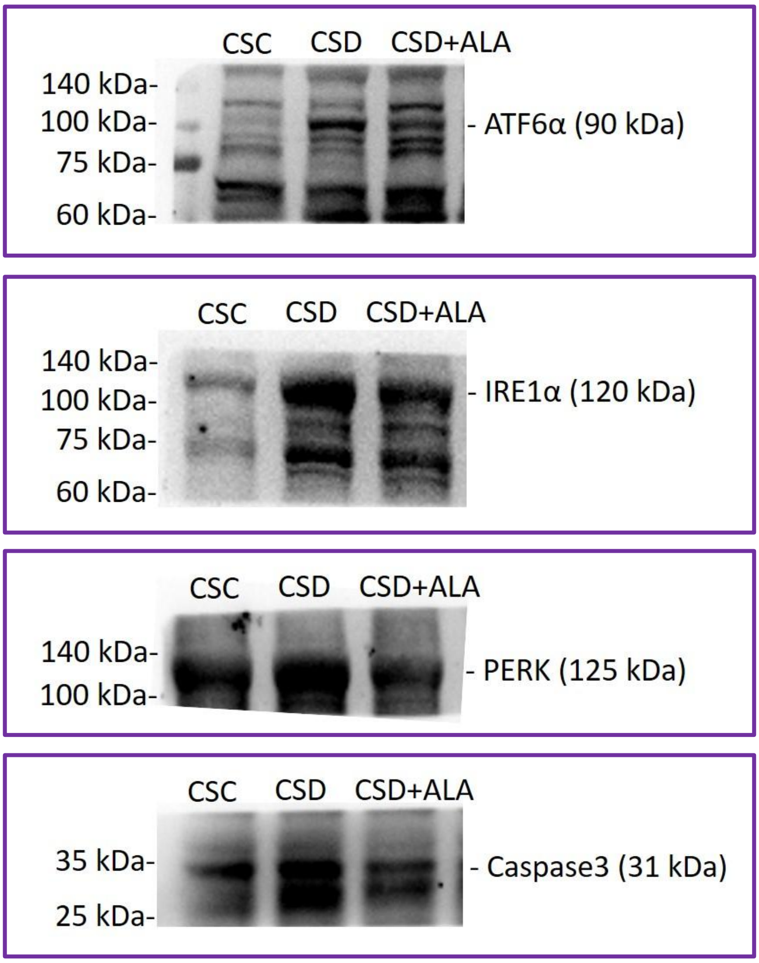

CSC
CSD
CSD+ALA
140 kDa-
100 kDa-
75 kDa-
60 kDa-
- ATF6α (90 kDa)
CSC
CSD
CSD+ALA
140 kDa-
100 kDa-
75 kDa-
60 kDa-
- IRE1α (120 kDa)
CSC
CSD
CSD+ALA
140 kDa-
100 kDa-
- PERK (125 kDa)
CSC
CSD
CSD+ALA
35 kDa-
25 kDa-
- Caspase3 (31 kDa)
