# Supplementary material for: Manifold hepatoprotective actions of α-lipoic acid on metabolic function through redox regulation, inflammatory modulation, and anti-apoptosis after chronic sleep-deprived injury
Source: Front Nutr. 2026 Jan 22;12:1679494. doi: 10.3389/fnut.2025.1679494 (PMC12872533; doi:10.3389/fnut.2025.1679494)
Supplement: Supplementary file 2 [file Image_2.pdf]

Manifold Hepatoprotective Actions of α-Lipoic Acid on Metabolic Function through Redox Regulation, Inflammatory Modulation, and Anti-apoptosis after Chronic Sleep-deprived Injury

HUNG-MING CHANG[1,2#], HSING-CHUN LIN[3,4#], TING-YI RENN[1], YU-CHENG LIU[5]
KAI-JUNG YEN[6], CHIH-KAI LIAO[6], MARIA A TIKHONOVA[7],
TAMARA G AMSTISLAVSKAYA[7], SANDEEP KUMAR SINGH[8], LI-YOU CHEN[6,9]*

Supplementary Data

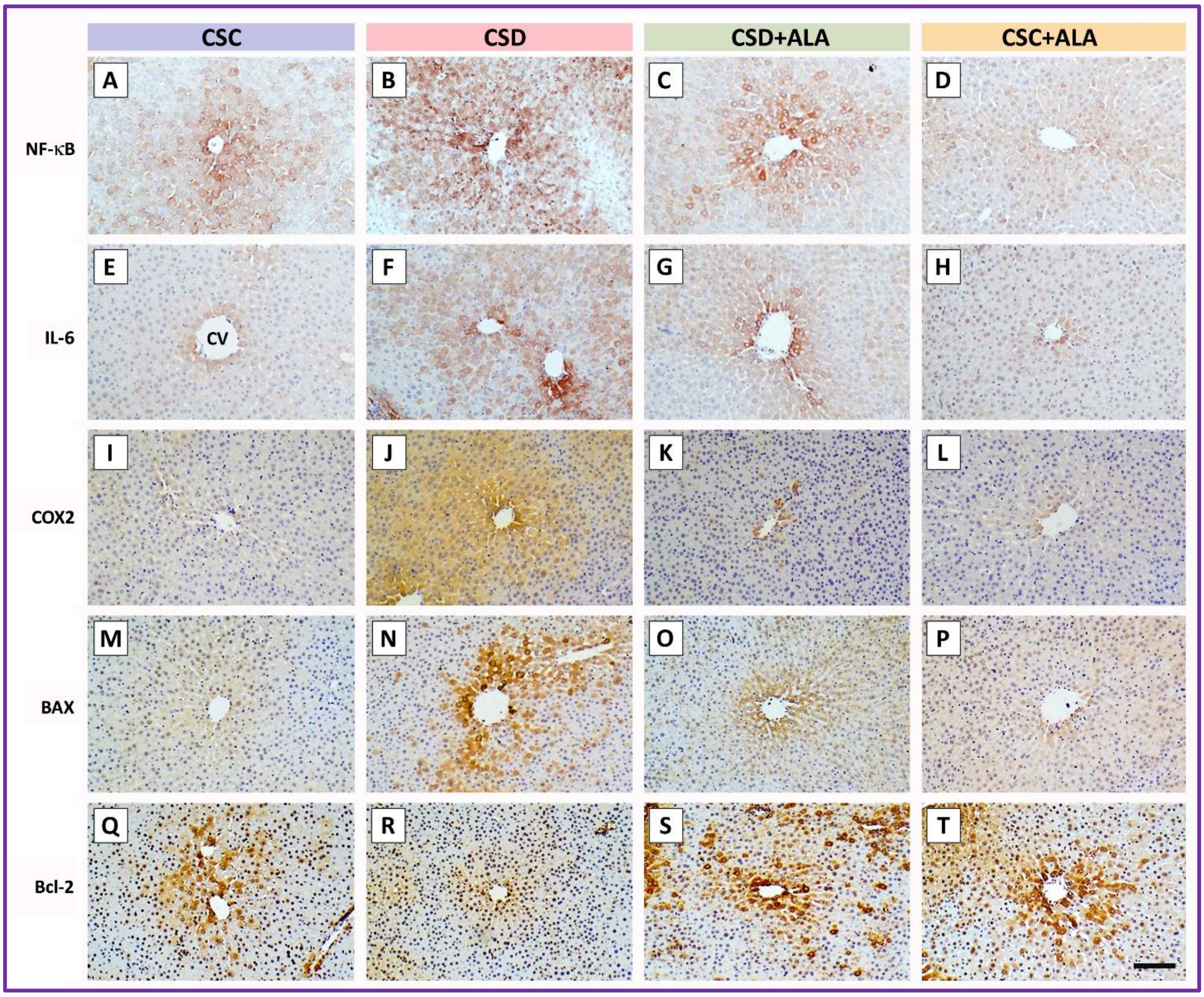

Supplementary Figure 1

Fig. S1. Photomicrographs (A-T) showed the immunohistochemical staining of NF-κB (A-D), IL-6 (E-H), COX2 (I-L), BAX (M-P), and Bcl-2 (Q-T) in the liver of control (CSC), chronic sleep deprivation (CSD), chronic sleep deprivation with alpha lipoic acid treatment (CSD+ALA), and control with alpha lipoic acid treatment (CSC+ALA) rats. Note that

exogenous supplementation with ALA during chronic sleep deprivation significantly reduced the expression of inflammatory factors (NF-κB, IL-6, and COX2) (C,G,K) and the pro-apoptotic factor (BAX) (O), while successfully increasing the immunoreactivity of the anti-apoptotic protein (Bcl-2) (S). However, in ALA-only group under normal sleep conditions (i.e. CSC+ALA group), no obvious changes of the immuno-expression were detected as compared with that of the control ones (D,H,L,P,T). These results demonstrate that ALA along does not alter inflammatory and apoptotic factors, which indicates that ALA itself (at least at the current dose) would exert only minimal effects, if any, on modulating the baseline hepatic or metabolic functions. CV: Central Vein. Scale bar = 100 μm.
